# Combined Linkage and Association Studies Show that HLA Class II Variants Control Levels of Antibodies against Epstein-Barr Virus Antigens

**DOI:** 10.1371/journal.pone.0102501

**Published:** 2014-07-15

**Authors:** Vincent Pedergnana, Laurène Syx, Aurélie Cobat, Julien Guergnon, Pauline Brice, Christophe Fermé, Patrice Carde, Olivier Hermine, Catherine Le- Pendeven, Corinne Amiel, Yassine Taoufik, Alexandre Alcaïs, Ioannis Theodorou, Caroline Besson, Laurent Abel

**Affiliations:** 1 Laboratory of Human Genetics of Infectious Diseases, Necker Branch, Institut National de la Santé et de la Recherche Médicale, Paris, France; 2 University Paris Descartes, Sorbonne Paris Cité, Imagine Institute, Paris, France; 3 Laboratory of Immunity and Infection, Institut National de la Santé et de la Recherche Médicale UMR-S 945, Université Pierre et Marie Curie Université Paris 6, Groupe Hospitalier Pitié-Salpêtrière, Assistance Publique-Hôpitaux de Paris, Paris, France; 4 Service d’Onco-Hématologie, Hôpital Saint-Louis, Paris, France; 5 Department of Medicine, Hôpital Gustave Roussy, Villejuif, France; 6 Service d’Hématologie adultes, Hôpital Necker, Assistance Publique-Hôpitaux de Paris, Paris, France; 7 Laboratoire de Virologie, Hôpital Tenon, Assistance Publique-Hôpitaux de Paris, Paris, France; 8 Assistance Publique-Hôpitaux de Paris, CHU Bicetre, Service d’Hématologie et Immunologie Biologiques, Le Kremlin-Bicetre, France, and Université Paris Sud, Faculte de Médecine, Le Kremlin-Bicetre, France, and Institut National de la Santé et de la Recherche Médicale U1012, Le Kremlin-Bicetre, France; 9 St Giles Laboratory of Human Genetics of Infectious Diseases, Rockefeller Branch, The Rockefeller University, New York, New York, United States of America; University of Nebraska – Lincoln, United States of America

## Abstract

Over 95% of the adult population worldwide is infected with Epstein-Barr virus (EBV). EBV infection is associated with the development of several cancers, including Hodgkin lymphoma (HL). Elevated levels of anti-EBV antibodies have been associated with increased risk of HL. There is growing evidence that genetic factors control the levels of antibodies against EBV antigens. Here, we conducted linkage and association studies to search for genetic factors influencing either anti-viral capsid antigen (VCA) or anti-Epstein Barr nuclear antigen-1 (EBNA-1) IgG levels in a unique cohort of 424 individuals of European origin from 119 French families recruited through a Hodgkin lymphoma (HL) patient. No major locus controlling anti-VCA antibody levels was identified. However, we found that the HLA region influenced anti-EBNA-1 IgG titers. Refined association studies in this region identified a cluster of HLA class II variants associated with anti-EBNA-1 IgG titers (e.g. p = 5×10–5 for rs9268403). The major allele of rs9268403 conferring a predisposition to high anti-EBNA-1 antibody levels was also associated with an increased risk of HL (p = 0.02). In summary, this study shows that HLA class II variants influenced anti-EBNA-1 IgG titers in a European population. It further shows the role of the same variants in the risk of HL.

## Introduction

Over 95% of the adult population worldwide is infected with Epstein-Barr virus (EBV) [1]. Primary EBV infection is mostly asymptomatic during childhood, but may be associated with infectious mononucleosis if it occurs later in life. The virus is transmitted in saliva. It then infects the epithelial cells and B lymphocytes of the oropharynx and spreads throughout the lymphoid tissues, remaining latent in memory B cells [1]. This latent phase is usually asymptomatic, but EBV is associated with several cancers, including nasopharyngeal carcinoma, Burkitt lymphoma and Hodgkin lymphoma (HL) [1]. The EBV antigens classically used for serological testing include Epstein-Barr nuclear antigen-1 (EBNA-1) and viral capsid antigen (VCA). EBNA-1 is expressed during latent infection, reflecting a history of infection, and VCA is expressed during lytic infection, thus reflecting reactivation. Recent studies have provided evidence for a role of genetic factors in the control of both anti-VCA and anti-EBNA-1 immunoglobulin (Ig) G levels. High heritability has been reported for both anti-VCA IgG levels in three familial samples of different origins [2], and for anti-EBNA-1 IgG levels in a Mexican American sample [3]. In this second study, a combined linkage and association study showed that at least two separate loci within the HLA region influenced anti-EBNA-1 antibody production. We carried out linkage and association studies to search for genetic factors influencing either anti-VCA or anti-EBNA-1 IgG levels in a French familial sample.

## Materials and Methods

### Families and serological methods

Families of European origin were recruited through index cases of HL as described elsewhere [2], [4]. Briefly, HL cases were recruited from three haematology units in the Paris area. Patients were included at diagnosis or whilst in complete remission, at least one year after diagnosis. Inclusion criteria were age between 15 and 35 years and negative serological tests for HIV at diagnosis. First-degree relatives (parents and siblings) of the patients were asked to participate in the study. Anti-VCA IgG and anti-EBNA-1 IgG titers were determined with ELISA kits (ImmunoWELL) and are expressed in international units per liter [2], [5]. Serological measurements were performed in the same laboratory, with ELISA kits from the same batch, by the same technician. Anti-VCA IgG and anti-EBNA-1 IgG titers were determined twice in 72 and 78 individuals, respectively. The intraclass correlation was high and similar for the two measures, with a value of 0.95 and 0.93, for anti-VCA and anti-EBNA-1 antibody levels, respectively. Positive serological results for EBV were defined as having either an anti-VCA IgG titer or an anti-EBNA-1 IgG titer above the corresponding threshold established by the manufacturer of the ELISA kits, ie only individuals who are negative for both tests were considered as having a negative EBV serology. IgG levels were determined by nephelometry. This study was approved by the French Consultative Committee for Protecting Persons in Biomedical Research (CCPPRB) of Paris Necker and Kremlin-Bicêtre. Written informed consent was obtained from all study participants. Written informed consent was obtained from the next of kin, caretakers, or guardians on behalf of the minors/children enrolled in your study.

### Genotyping

**Figure 1:**
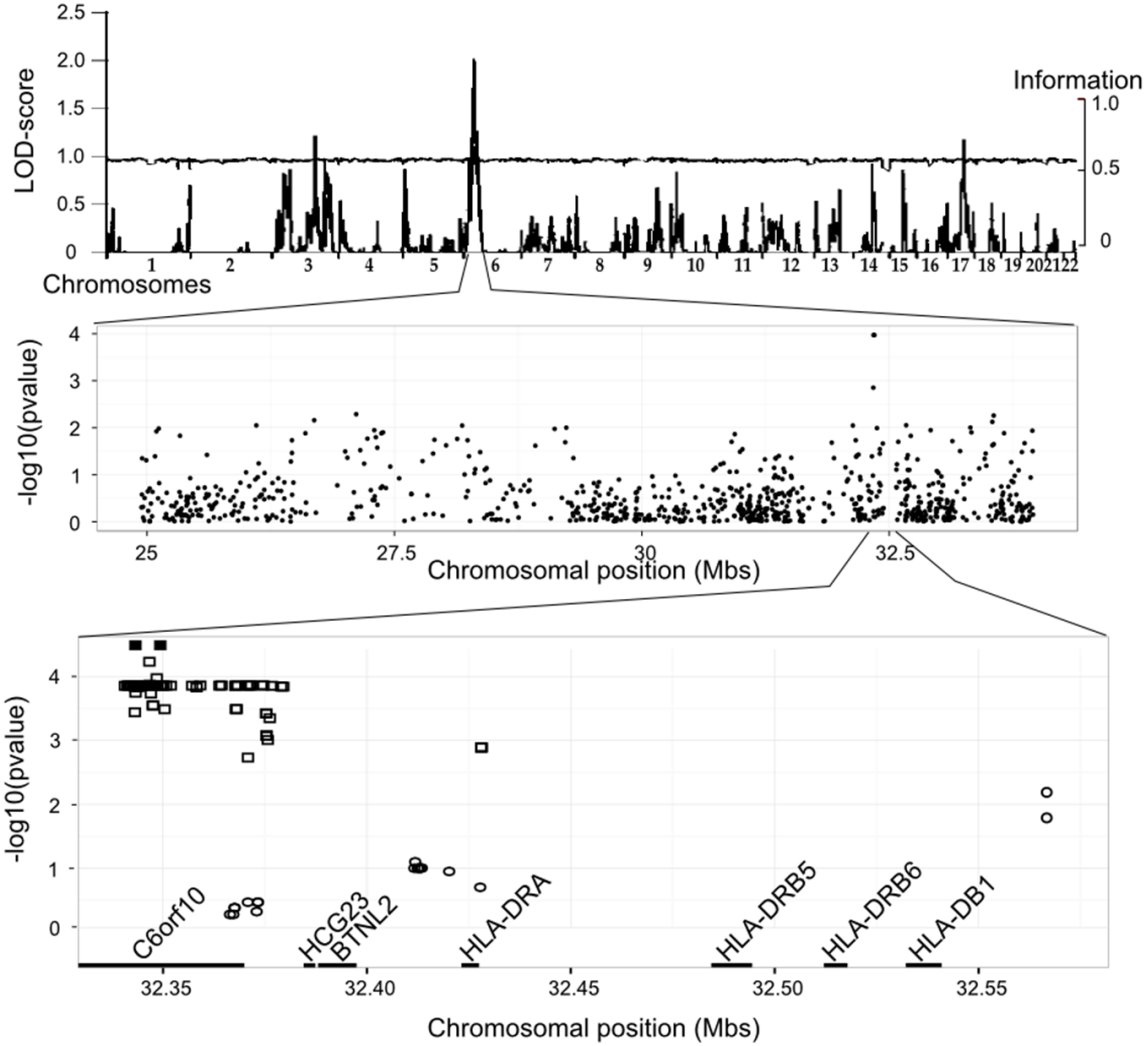
Genome-wide linkage analysis and association study of anti-EBNA-1 IgG levels in the linked 6p region. Top panel: Multipoint LOD score (left y-axis) and information content (right y-axis) are plotted along the 22 autosomes (x axis). Mid panel: Results of association study for 824 SNPs located in the 90% confidence linkage interval are given as −log_10_ (pvalue) (y-axis) and plotted against SNP positions on chromosome 6 (x-axis in megabases). Bottom panel: Association results as −log_10_ (pvalue) (y axis) with 1) the cluster of SNPs in strong linkage disequilibrium (r^2^>0.8) with rs9268403 (squares), and 2) the SNPs reported in [3](circles); the two genotyped SNPs are shown under dominant model (plain symbol), and all the others SNPs are imputed and shown under additive model (open symbols); chromosome 6 position of SNPs and genes in the region are given on the x axis (in megabases).

Genomic DNA was extracted from blood samples, using the QIamp DNA blood Mini Kit (Qiagen, Hilden, Germany). Genotyping was performed with the Illumina HumanCytoSNP12v2.1 Panel, containing 299,140 single nucleotide polymorphisms (SNPs). Quality control (QC) for the data was performed with PLINK software (http://pngu.mgh.harvard.edu/~purcell/plink/). For genetic markers, we applied the following QC criteria: call rate ≥95%, minor allele frequencies (MAF) ≥0.05, *p*-value for Hardy-Weinberg equilibrium ≥10^−5^ and no Mendelian errors. In total, 255,341 high-quality SNPs were considered for further analysis. For individuals, we excluded three samples based on a call rate <98% and/or the presence of Mendelian errors, resulting in the limitation of the analysis to 414 subjects. For linkage analyses, we selected 40,783 common SNPs (MAF≥0.2) displaying low levels of linkage disequilibrium (LD) (r^2^≤0.2). Information content (IC) was very high across all autosomes (mean = 0.964), with more than 90% of the genome having an IC>95% (Figure 1). For association studies, all SNPs with a MAF≥0.05 in the linked regions of interest were used.

### Statistical analysis

We first investigated linkage for anti-VCA and anti-EBNA-1 IgG levels, using the new maximum-likelihood binomial (MLB) model-free method for quantitative traits (nMLB-QTL v.3.0) [6], [7]. This approach considers the sibship as a whole and makes no assumptions about the distribution of the phenotype. The linkage test is asymptotic, as a 50∶50 mixture of χ^2^ distributions with 0 and 1 degree of freedom, and the result can be expressed as a lod-score with the same distribution as a classical model-based lod-score. We estimated the power of our sample to detect suggestive linkage as defined by a lod-score >2.2 [8] by conducting a simulation study. We generated phenotypes under different underlying genetic models, and keeping the familial structure (and the number of individuals) as well as the genotyping data as they were really observed in our sample. As genetic models, we considered a diallelic locus (minor allelic frequency of 0.45) influencing a quantitative phenotype with an additive effect and leading to a trait heritability ranging from 0.3 to 0.5. We generated 100 replicates for each heritability value and we performed linkage analysis in each replicate. The power of our sample to observe a suggestive linkage was 37%, 50%, 66%, 83%, and 97% for heritability values of 0.3, 0.35, 0.4, 0.45, and 0.5, respectively.

Family-based association tests (FBATs) were carried out on linked regions with FBAT software, version 2.0.3, to test for association in the classical transmission disequilibrium test with the empirical variance, to account for linkage [9]. Pairwise LD measures (r^2^) were calculated across the genome with HAPLOVIEW [10]. Haplotypes of genotyped variants in the linkage region were phased with ShapeIT [11] and untyped variants were imputed with Impute2 v.2.1.2 software (http://mathgen.stats.ox.ac.uk/impute_v2.html), based on the 1000 Genomes phase I reference panel. Variants with a MAF≥0.05 were included in subsequent analyses if they had an imputation information score greater than 0.6. We also performed a specific imputation for HLA class II alleles using HIBAG, the method described by Zheng et al [12]. Family-based association tests (FBATs) for high-quality imputed variants were performed by a new analytical approach (FBATdosage) accounting for genotype uncertainty through the use of the expected allele count, also known as allele dosage, in the general FBAT framework [13].

## Results

### Study sample

The overall seroprevalence of EBV in this sample was 98% (417/424 subjects). None of the seven subjects negative for anti-VCA antibodies were positive for anti-EBNA-1 antibodies, while 379 out of the 417 (91%) subjects with positive anti-VCA antibodies had also positive anti-EBNA-1 antibodies. The distribution of the log-transformed anti-VCA and anti-EBNA-1 IgG antibody levels in the EBV-positive subjects is shown in Figure S2, and the correlation between the two measures was 0.37. Our final sample included 414 genotyped EBV-positive subjects from 119 nuclear families. The mean age of the individuals was 44 years (range: 14.5–87.4 years), and the male-to-female ratio was 0.82. For linkage analyses, 86 families were informative, i.e. with at least two available offspring. The phenotypes of interest were anti-VCA and anti-EBNA-1 IgG levels, expressed in international units per liter. In our sample, the distributions of anti-VCA and anti-EBNA-1 IgG titers were strongly skewed to the right. We therefore used the decimal logarithm of these traits in all our analyses. Regression analyses showed no significant effect of age or sex on either of these antibody phenotypes. There was a strong correlation between anti-VCA IgG and total IgG levels (*ρ* = 0.33, *p* = 10^−10^), but only a non significant trend towards a correlation between total IgG levels and anti-EBNA-1 IgG (*ρ* = 0.1, *p* = 0.053).

### Anti-VCA antibodies

Linkage analysis on crude data for anti-VCA IgG levels indicated a suggestive peak, with a lod-score of 3.09, on chromosome 2q33.3 (*p* = 8×10^−5^ at position 206.65 Mbs; IC = 0.961) (Figure S1a). We then carried out a linkage analysis in which anti-VCA antibody levels were adjusted for total IgG level, as we observed a strong correlation between these two traits. The initially identified peak on chromosome 2q33.3 was much smaller, with a lod-score of 1.43 (Figure S1b). No suggestive linkage peaks were identified in other regions. These results suggest that the initial peak was not specific for anti-VCA IgG level, instead probably being largely due to linkage with total IgG levels. No further association analyses were carried out on anti-VCA antibodies.

### Anti-EBNA-1 antibodies

**Figure 2:**
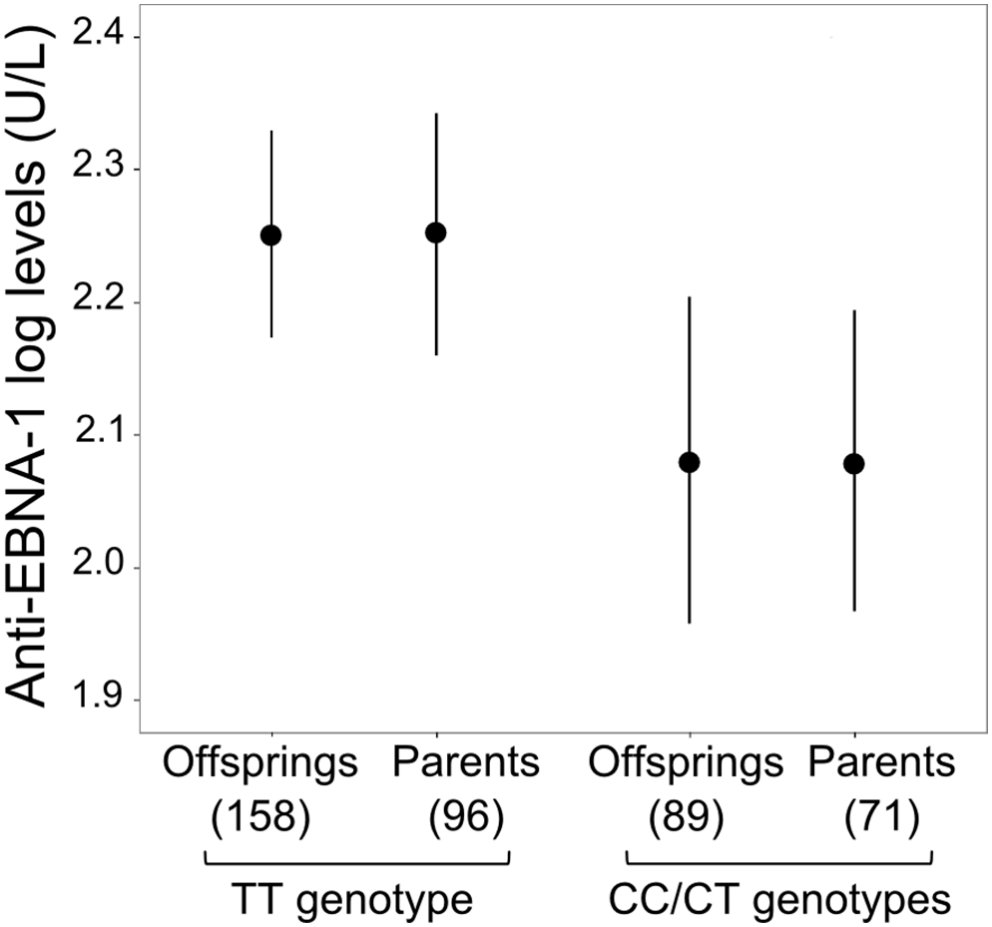
Anti-EBNA-1 IgG distributions among offspring and parent samples as a function of their genotype. Mean titers (black circles) with 95% confidence interval for anti-EBNA-1 IgG levels are shown as a function of the genotype at rs9268403 under a dominant model for the minor allele C (TT vs CC/CT).

A single linkage peak was observed in the HLA region on chromosome 6p21.3, with a lod-score of 2.02 (*p* = 10^−3^ at position 29.79 Mbs; IC = 0.965) (Figure 1). This result was not substantially modified by the adjustment of EBNA-1 IgG levels for total IgG levels (lod-score = 2.12, data not shown), as expected given the lack of correlation between these two traits. This peak overlaps the linkage region recently identified for anti-EBNA-1 antibodies [3], rendering this result particularly interesting for further association studies. The 90% confidence interval, corresponding to a 1 LOD decrease from the linkage peak, defined a 9 Mbs region extending from 24.94 to 33.95 Mb. The region of interest contains 824 genotyped SNPs with a MAF≥0.05, which were tested for association with anti-EBNA-1 antibody levels (Figure 1). Two SNPs, rs9268403 and rs9268454, in perfect LD (r^2^ = 1), were found to be associated with anti-EBNA-1 IgG levels (p = 10^−4^) and, for the sake of simplicity, we present only the results for rs9268403, as identical results were obtained for rs9268454. As the frequency of the minor allele C of rs9268403 was low (MAF = 0.23), with few CC homozygotes, we also performed the analysis with a dominant model for the C allele, in which a more significant association was detected (*p* = 5×10^−5^). Offspring homozygous for the major allele (T) of rs9268403 had higher anti-EBNA-1 antibody levels than CT/CC subjects (Figure 2). As the association analysis was based on familial tests, we also investigated anti-EBNA-1 antibody levels as a function of rs9268403 genotype in the 167 available parents, providing additional information independent of the familial transmission of alleles. We observed the same association, with TT parents having significantly higher anti-EBNA-1 IgG levels (p = 0.033) than CT/CC parents (Figure 2). These findings provide strong support for the association of rs9268403 with anti-EBNA-1 antibody levels.

We then carried out imputation in the linkage region, using the 1000 Genomes reference panel. In total, 51,030 SNPs could be imputed with imputation information scores≥0.6 and a MAF≥0.05, including 84 SNPs in strong LD (r^2^≥0.8) with rs9268403 (Table S1). An association study with the imputed SNPs based on FBAT-dosage confirmed that the best signals were obtained with the cluster of SNPs in strong LD with rs9268403. This cluster of 86 SNPs (including rs9268403 and rs9268454) is located between 32.34 Mb and 32.43 Mb, in a region overlapping the genes *C6orf10, HCG23* and *BTNL2*, close to *HLA-DRA* (Figure 1). This region also contains most of the SNPs reported in a previous study [3] (Figure 1). We were able to replicate the effect of rs477515 (*p* = 0.017, r^2^ with rs9268403 = 0.62 in European population) and rs2516049 (*p* = 0.007, r^2^ with rs9268403 = 0.74), the two most significant SNPs of this previous study, located ∼200 kb from rs9268403 (Figure 1). In both studies, the minor allele was associated with lower anti-EBNA-1 IgG levels. The other SNPs reported in the previous study [3] were not significantly associated with anti-EBNA-1 IgG levels in our sample. The previous study was conducted in Mexican American subjects, and allele frequencies and LD patterns differ between the MEX and CEU HapMap populations (Table S1), potentially accounting, at least in part, for the observed difference between the two studies.

We then investigated whether our associated SNPs had been described as expression quantitative trait loci (eQTLs), using data from the GTEx eQTL database (http://www.ncbi.nlm.nih.gov/gtex/GTEX2/gtex.cgi), which explores eQTL loci in lymphoblastoid cells. Eight of the 86 SNPs from our cluster had been reported to be significantly associated with mRNA levels for HLA-DQA1, HLA-DQA2, and HLA-DQB1 (Table S2). One of the most significant findings was the association between rs9268403 and HLA-DQA1 expression. The 27 SNPs reported in the previous study [3] were eQTLs for the same three genes and for HLA-DRB5 (Table S2). Interestingly, rs2516049, the SNP we were able to replicate in our sample, had also been identified as an eQTL for HLA-DQA1. Among these HLA class II loci of interest we were able to impute reliable four digits HLA-DQA1 and HLA-DQB1 alleles [12] in ∼85% of our study subjects. We observed moderate LD of rs9268403 with three HLA- DQA1 (*0201, r2 = 0.16; *0301, r2 = 0.17;*0303, r2 = 0.18), and two HLA-DQB1 (*0202, r2 = 0.31; *0302, r2 = 0.18) alleles. However, none of these HLA alleles provided significant association with anti-EBNA-1 antibody levels.

Finally, we also made use of our sample recruited through HL probands to test the association of rs9268403 with HL in FBAT. The major allele (T) of rs9268403, which is associated with higher levels of anti-EBNA-1 antibodies, was also significantly associated (*p* = 0.02) with a higher risk of HL. Several HLA variants had already been shown to be associated with HL in genome-wide association studies (GWAS) [14] and five were reported in the Phenotype-Genotype Integrator database (http://www.ncbi.nlm.nih.gov/gap/phegeni) (Table S2). Two of these variants were found to be in strong LD with rs9268403, this LD being strongest for rs2395185 (r^2^ with rs9268403 = 0.52).

## Discussion

In this study, we investigated the genetic control of anti-VCA and anti-EBNA-1 antibody levels. We found no evidence for linkage with a locus specifically controlling anti-VCA IgG levels. However, it should be noted that our sample had limited power to detect suggestive linkage with a locus providing a trait heritability lower than 0.35 (see methods). In contrast, we were able to replicate the implication of the HLA region in the regulation of anti-EBNA-1 IgG levels. Association studies on this region provided strong evidence for a role of rs9268403, which is part of a large cluster of SNPs in strong LD.

We were also able to replicate the implication of the two most significant SNPs, rs2516049 and rs477515, identified in a previous study [3], both of which are in substantial LD with rs9268403 in European populations (r^2^>0.6). Both rs9268403 and rs2516049 have been identified as eQTLs for the same HLA class II genes, including HLA-DQA1 in particular. We could perform imputation for HLA-DQA1 and HLA-DQB1 alleles, and none of them showed a significant association with anti-EBNA-1 antibody levels. However our SNP data are not optimal to impute HLA class II alleles, and a detailed investigation of their effect will need further in-depth studies with the complete molecular genotyping of HLA class II variants.

Finally, the major allele of rs9268403 predisposing to high levels of anti-EBNA-1 antibodies was also associated with an increased risk of HL. This result is consistent with the known association of elevated levels of anti-EBNA antibodies with increased risk of HL [15]. Interestingly, rs9268403 was also found to be in LD with at least one SNP associated with HL in GWAS. Further studies are required to determine whether the genetic control of anti-EBNA-1 antibody levels can account, at least in part, for the genetic control of HL, and to identify precisely the HLA class II variants involved. This work will be particularly challenging, due to the high level of LD reported for this HLA region.

## Supporting Information

Figure S1 **Genome-wide linkage model-free analysis of anti-VCA IgG levels.** Multipoint LOD-score (left y-axis) and information content (right y-axis) are plotted along the 22 autosomes (x axis). a) Analysis before total IgG adjustment. b) Analysis after total IgG adjustment.
(DOCX)

Figure S2 **Distribution of anti-VCA (a) and anti-EBNA-1 (b) IgG antibody levels in the 417 EBV-positive subjects.**
(DOCX)

Table S1 **Comparison of minor allele frequency (MAF) and linkage disequilibrium (LD) between European (CEU) and Mexican (MEX) populations from HapMap project among (top panel) the 83 SNPs in LD >0.8 with rs9268403, and (bottom panel) the 21 SNPs of a previous study (1).** All the SNPs but rs9268403 and rs9268454 are imputed, and table also presents the results of association with anti-EBNA1 IgG levels under an additive model (p-value).
(DOCX)

Table S2 **Association between SNPs and gene expression.** This table shows eQTL gene, associated p-value and LD with rs9268403 of SNPs which are reported in the GTEx eQTL database (http://www.ncbi.nlm.nih.gov/gtex/GTEX2/gtex.cgi) with a p-value<10^−5^, and belong to one of these three categories: SNPs of supplementary table 1 identified by our present analysis (top panel), or by the previous study of Rubicz et al (3) (mid panel), and SNPs reported as associated with Hodgkin lymphoma (HL) in the Phenotype-Genotype Integrator database ((http://www.ncbi.nlm.nih.gov/gap/phegeni) (bottom panel).
(DOCX)
