# Supplementary figures and images for: Combined Linkage and Association Studies Show that HLA Class II Variants Control Levels of Antibodies against Epstein-Barr Virus Antigens
Source: PLoS One. 2014 Jul 15;9(7):e102501. doi: 10.1371/journal.pone.0102501 (PMC4099326; doi:10.1371/journal.pone.0102501)

**Figure S1**

***
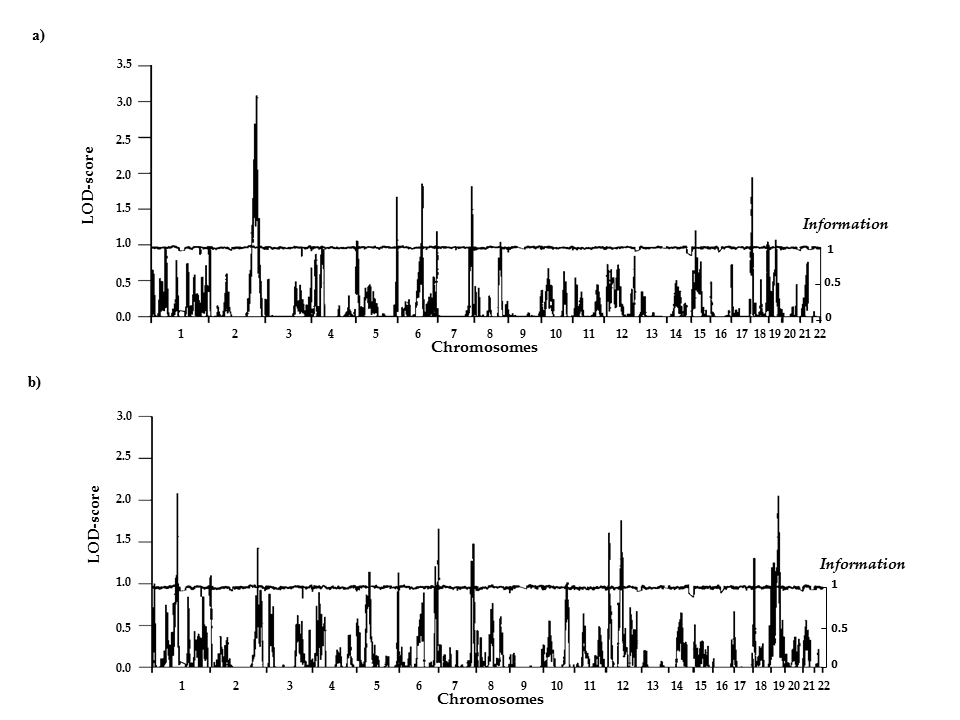
***

Supplement: Figure S1 — Genome-wide linkage model-free analysis of anti-VCA IgG levels. Multipoint LOD-score (left y-axis) and information content (right y-axis) are plotted along the 22 autosomes (x axis). a) Analysis before total IgG adjustment. b) Analysis after total IgG adjustment. (DOCX) [file pone.0102501.s001.docx]

**Figure S2**


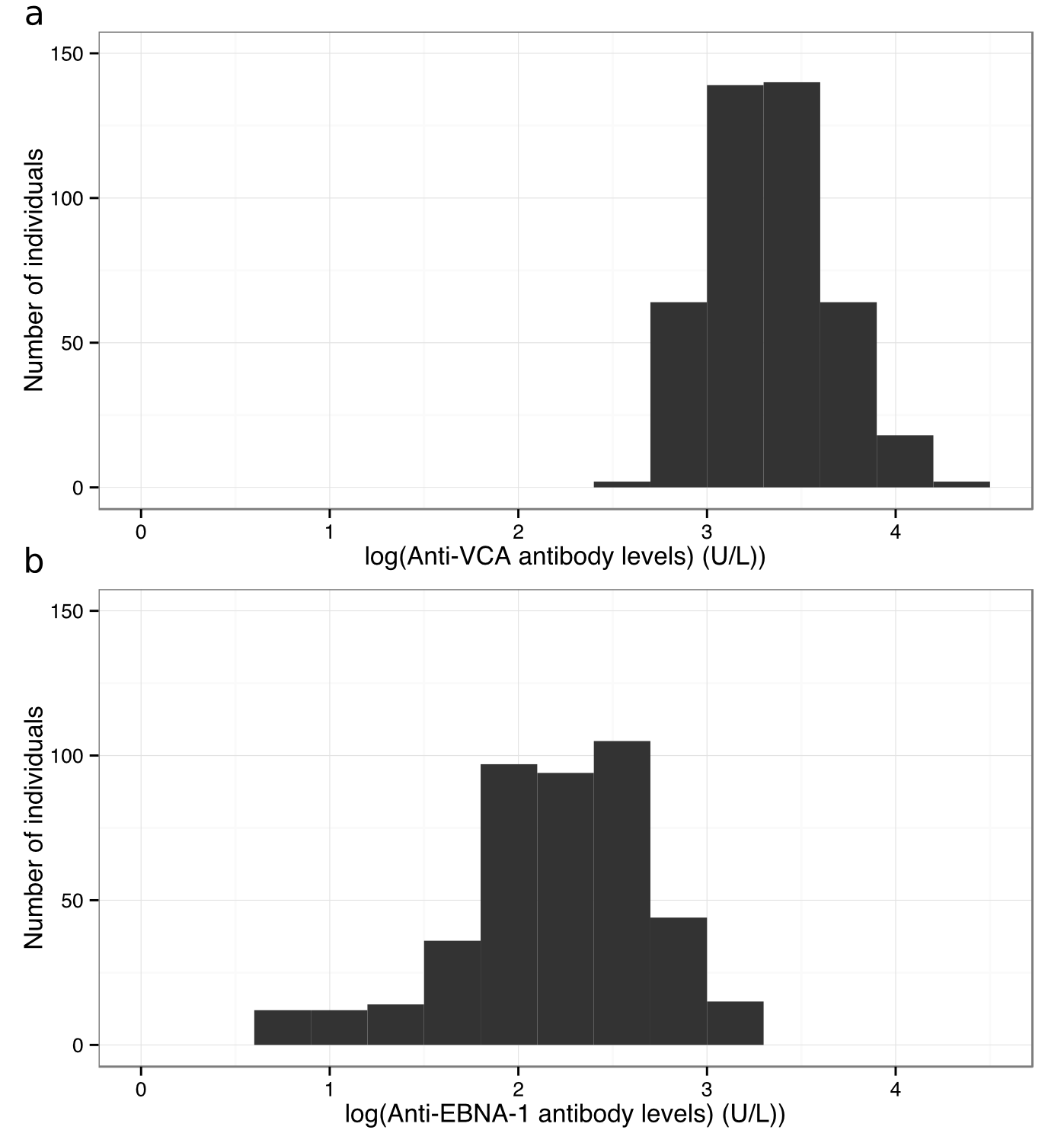

Supplement: Figure S2 — Distribution of anti-VCA (a) and anti-EBNA-1 (b) IgG antibody levels in the 417 EBV-positive subjects. (DOCX) [file pone.0102501.s002.docx]
