# Supplementary material for: Combined Linkage and Association Studies Show that HLA Class II Variants Control Levels of Antibodies against Epstein-Barr Virus Antigens
Source: PLoS One. 2014 Jul 15;9(7):e102501. doi: 10.1371/journal.pone.0102501 (PMC4099326; doi:10.1371/journal.pone.0102501)
Supplement: Table S1 — Comparison of minor allele frequency (MAF) and linkage disequilibrium (LD) between European (CEU) and Mexican (MEX) populations from HapMap project among (top panel) the 83 SNPs in LD >0.8 with rs9268403, and (bottom panel) the 21 SNPs of a previous study (1). All the SNPs but rs9268403 and rs9268454 are imputed, and table also presents the results of association with anti-EBNA1 IgG levels under an additive model (p-value). (DOCX) [file pone.0102501.s003.docx]

**Table S1**

| SNP | Location on chrom 6 (bps) | Minor Allele | MAF in CEU | MAF in MEX | p-value | LD (r²) with rs9268403 in Europe | LD (r²) with rs9268403 in Mexico |
| --- | --- | --- | --- | --- | --- | --- | --- |
| 83 SNPs in LD > 0.8 | | | | | | | |
| rs9268403 | **32341473** | C | **0.223** | **0.346** | **1.39E-04** | **1** | **1** |
| rs9268454 | **32349711** | G | **0.223** | **0.346** | **1.39E-04** | **1** | **1** |
| rs9268400 | 32340654 | A | 0.223 |  | 1.40E-04 | 1 |  |
| rs9268401 | 32341318 | G | 0.223 |  | 1.39E-04 | 1 |  |
| rs9268404 | 32341719 | T | 0.223 | 0.346 | 1.39E-04 | 0.928 | 1 |
| rs9268405 | 32342087 | T | 0.223 |  | 1.39E-04 | 0.964 |  |
| rs9268406 | 32342129 | T | 0.223 |  | 1.39E-04 | 1 |  |
| rs9268407 | 32342166 | C | 0.223 |  | 1.39E-04 | 1 |  |
| rs9268409 | 32342639 | C | 0.223 |  | 1.39E-04 | 1 |  |
| rs9268412 | 32343018 | A | 0.223 |  | 1.39E-04 | 1 |  |
| rs9268413 | 32343095 | C | 0.214 |  | 3.63E-04 | 0.928 |  |
| rs9268414 | 32343096 | A | 0.218 |  | 1.46E-04 | 1 |  |
| rs9268415 | 32343267 | C | 0.225 |  | 1.80E-04 | 1 |  |
| rs9268416 | 32343384 | G | 0.223 |  | 1.39E-04 | 1 |  |
| rs9268417 | 32343616 | G | 0.223 |  | 1.39E-04 | 0.963 |  |
| rs9268418 | 32343686 | C | 0.223 |  | 1.39E-04 | 1 |  |
| rs9268420 | 32343796 | T | 0.223 |  | 1.39E-04 | 0.963 |  |
| rs9268421 | 32343869 | G | 0.221 |  | 1.44E-04 | 0.826 |  |
| rs9268422 | 32343873 | A | 0.221 |  | 1.44E-04 | 0.928 |  |
| rs9268423 | 32343882 | T | 0.221 |  | 1.44E-04 | 1 |  |
| rs9268424 | 32343985 | C | 0.223 |  | 1.39E-04 | 1 |  |
| rs9268425 | 32344376 | T | 0.223 |  | 1.39E-04 | 1 |  |
| rs9268426 | 32344731 | G | 0.223 |  | 1.39E-04 | 1 |  |
| rs9268428 | 32344973 | T | 0.223 | 0.346 | 1.39E-04 | 1 | 1 |
| rs9268429 | 32345052 | G | 0.223 | 0.346 | 1.39E-04 | 1 | 1 |
| rs2894252 | 32345443 | T | 0.223 | 0.346 | 1.39E-04 | 1 | 1 |
| rs9268431 | 32345514 | G | 0.223 |  | 1.39E-04 | 1 |  |
| rs9268432 | 32345667 | G | 0.223 |  | 1.39E-04 | 1 |  |
| rs9268436 | 32346637 | G | 0.222 |  | 1.41E-04 | 1 |  |
| rs9268437 | 32346662 | T | 0.204 |  | 5.87E-05 | 0.963 |  |
| rs9268439 | 32346701 | A | 0.223 |  | 1.39E-04 | 0.963 |  |
| rs1980495 | 32346794 | C | 0.223 |  | 1.34E-04 | 1 |  |
| rs1980494 | 32347016 | G | 0.229 |  | 1.85E-04 | 0.893 |  |
| rs8180659 | 32347280 | T | 0.223 |  | 1.39E-04 | 1 |  |
| rs8180664 | 32347490 | T | 0.223 |  | 1.39E-04 | 1 |  |
| rs8180673 | 32347532 | T | 0.210 |  | 2.83E-04 | 1 |  |
| rs8180672 | 32347533 | G | 0.210 |  | 2.83E-04 | 1 |  |
| rs9268446 | 32347749 | G | 0.223 |  | 1.39E-04 | 0.964 |  |
| rs9268447 | 32347831 | C | 0.223 |  | 1.39E-04 | 1 |  |
| rs2395155 | 32347897 | T | 0.223 |  | 1.39E-04 | 1 |  |
| rs2395156 | 32348098 | A | 0.222 |  | 1.39E-04 | 1 |  |
| rs2395157 | 32348145 | G | 0.222 | 0.346 | 1.39E-04 | 1 | 1 |
| rs9268448 | 32348463 | G | 0.216 |  | 1.06E-04 | 0.928 |  |
| rs9268449 | 32348467 | A | 0.220 |  | 1.39E-04 | 0.893 |  |
| rs9268450 | 32348690 | A | 0.222 |  | 1.39E-04 | 1 |  |
| rs9268452 | 32349494 | G | 0.223 |  | 1.39E-04 | 1 |  |
| rs9268453 | 32349571 | C | 0.223 |  | 1.39E-04 | 1 |  |
| rs9268456 | 32349946 | A | 0.223 | 0.346 | 1.39E-04 | 1 | 1 |
| rs9268457 | 32350036 | G | 0.223 |  | 1.39E-04 | 0.963 |  |
| rs9268458 | 32350384 | A | 0.228 |  | 3.25E-04 | 1 |  |
| rs9268459 | 32350776 | A | 0.223 |  | 1.39E-04 | 1 |  |
| rs9268461 | 32351901 | A | 0.223 | 0.346 | 1.39E-04 | 1 | 1 |
| rs9268474 | 32357165 | C | 0.223 | 0.337 | 1.39E-04 | 1 | 0.958 |
| rs9268475 | 32358231 | T | 0.223 |  | 1.48E-04 | 1 |  |
| rs9268477 | 32359121 | A | 0.222 |  | 1.38E-04 | 1 |  |
| rs9268480 | 32363844 | T | 0.223 | 0.337 | 1.39E-04 | 1 |  |
| rs9268481 | 32364356 | G | 0.223 |  | 1.38E-04 | 1 |  |
| rs2294880 | 32367722 | G | 0.222 |  | 1.40E-04 | 1 |  |
| rs9268482 | 32367777 | T | 0.223 |  | 1.38E-04 | 1 |  |
| rs3817966 | 32367847 | C | 0.228 |  | 3.24E-04 | 1 |  |
| rs3817963 | 32368087 | C | 0.228 | 0.356 | 3.23E-04 | 1 | 0.878 |
| rs3817962 | 32368314 | A | 0.223 | 0.337 | 1.38E-04 | 1 | 0.958 |
| rs2076525 | 32370616 | C | 0.223 | 0.337 | 1.38E-04 | 1 | 0.958 |
| rs2076524 | 32370684 | G | 0.223 | 0.337 | 1.38E-04 | 1 | 0.958 |
| rs2076523 | 32370835 | C | 0.285 | 0.452 | 1.86E-03 | 0.806 | 0.336 |
| rs2076522 | 32371179 | C | 0.223 |  | 1.38E-04 | 1 |  |
| rs2076520 | 32371269 | T | 0.223 | 0.337 | 1.38E-04 | 1 | 0.958 |
| rs3793126 | 32371619 | G | 0.223 |  | 1.38E-04 | 1 |  |
| rs9268491 | 32374131 | G | 0.222 |  | 1.38E-04 | 0.964 |  |
| rs3763307 | 32374622 | T | 0.222 |  | 1.38E-04 | 1 |  |
| rs9268492 | 32375280 | G | 0.241 |  | 3.78E-04 | 1 |  |
| rs9268493 | 32375330 | A | 0.241 | 0.365 | 3.78E-04 | 1 | 0.841 |
| rs9268494 | 32375352 | C | 0.246 | 0.385 | 8.42E-04 | 1 | 0.772 |
| rs9268495 | 32375371 | T | 0.246 |  | 8.42E-04 | 1 |  |
| rs9268496 | 32375374 | A | 0.246 |  | 8.42E-04 | 1 |  |
| rs9268497 | 32375424 | A | 0.246 |  | 8.42E-04 | 1 |  |
| rs9268499 | 32375695 | A | 0.243 | 0.365 | 9.84E-04 | 1 | 0.841 |
| rs3763311 | 32376176 | T | 0.237 | 0.346 | 4.49E-04 | 1 | 0.917 |
| rs3763316 | 32376746 | T | 0.223 | 0.337 | 1.40E-04 | 1 | 0.958 |
| rs9268514 | 32378945 | A | 0.223 |  | 1.43E-04 | 0.928 |  |
| rs9268516 | 32379489 | T | 0.223 | 0.337 | 1.44E-04 | 1 | 0.958 |
| rs9268833 | 32428062 | T | 0.207 | 0.327 | 1.29E-03 | 0.821 | 0.325 |
| rs9268834 | 32428079 | A | 0.207 |  | 1.29E-03 | 0.821 |  |
| rs9268835 | 32428115 | A | 0.207 |  | 1.29E-03 | 0.821 |  |
|  |  |  |  |  |  |  |  |
|  |  |  |  |  |  |  |  |
| 21 SNPs of the previous study (1) | | | | | | | |
| rs10947261 | 32373232 | T | 0.068 | 0.221 | 3.41E-01 | 0.032 | 0.15 |
| rs10947262 | 32373312 | T | 0.068 | 0.221 | 3.41E-01 | 0.032 | 0.15 |
| rs204999 | 32109979 | G | 0.270 | 0.183 | 2.12E-01 | 0.003 | 0 |
| rs2213585 | 32413150 | G | 0.334 | 0.385 | 1.00E-01 | 0.279 | 0.203 |
| rs2213586 | 32413094 | A | 0.334 | 0.385 | 1.00E-01 | 0.279 | 0.203 |
| rs2227139 | 32413459 | G | 0.334 | 0.385 | 9.95E-02 | 0.279 | 0.203 |
| rs2239803 | 32411833 | C | 0.467 | 0.471 | 7.96E-02 | 0.593 | 0.32 |
| rs2294881 | 32367604 | C | 0.158 | 0.288 | 4.15E-01 | 0.123 | 0.215 |
| rs2294882 | 32367515 | C | 0.158 | 0.288 | 4.16E-01 | 0.123 | 0.215 |
| rs2294884 | 32367259 | G | 0.153 | 0.269 | 5.29E-01 | 0.123 | 0.195 |
| rs2516049 | 32570400 | C | 0.212 | 0.288 | 6.64E-03 | 0.739 | 0.425 |
| rs28362680 | 32370816 | A | 0.068 | 0.221 | 3.41E-01 | 0.027 | 0.15 |
| rs28362683 | 32372963 | A | 0.063 | 0.202 | 4.78E-01 | 0.032 | 0.134 |
| rs4248166 | 32366421 | C | 0.153 | 0.269 | 5.29E-01 | 0.123 | 0.195 |
| rs477515 | 32569691 | A | 0.258 | 0.288 | 1.67E-02 | 0.623 | 0.425 |
| rs652888 | 31851234 | G | 0.187 | 0.115 | 7.30E-01 | 0.13 | 0.04 |
| rs7192 | 32411646 | T | 0.334 | 0.385 | 1.00E-01 | 0.279 | 0.203 |
| rs7194 | 32412480 | G | 0.334 | 0.385 | 1.00E-01 | 0.279 | 0.203 |
| rs7195 | 32412539 | A | 0.334 | 0.385 | 1.00E-01 | 0.279 | 0.203 |
| rs7754768 | 32420179 | C | 0.364 | 0.385 | 1.12E-01 | 0.279 | 0.203 |
| rs9268832 | 32427789 | T | 0.347 | 0.356 | 1.99E-01 | 0.279 | 0.171 |
