# Supplementary material for: Combined Linkage and Association Studies Show that HLA Class II Variants Control Levels of Antibodies against Epstein-Barr Virus Antigens
Source: PLoS One. 2014 Jul 15;9(7):e102501. doi: 10.1371/journal.pone.0102501 (PMC4099326; doi:10.1371/journal.pone.0102501)
Supplement: Table S2 — Association between SNPs and gene expression. This table shows eQTL gene, associated p-value and LD with rs9268403 of SNPs which are reported in the GTEx eQTL database (http://www.ncbi.nlm.nih.gov/gtex/GTEX2/gtex.cgi) with a p-value<10−5, and belong to one of these three categories: SNPs of supplementary table 1 identified by our present analysis (top panel), or by the previous study of Rubicz et al (3) (mid panel), and SNPs reported as associated with Hodgkin lymphoma (HL) in the Phenotype-Genotype Integrator database ((http://www.ncbi.nlm.nih.gov/gap/phegeni) (bottom panel). (DOCX) [file pone.0102501.s004.docx]

**Table S2**

| **SNP** | **Position** | **eQTL** | **p-value** | **LD (r²)** |
| --- | --- | --- | --- | --- |
|  | | | | |
| **SNPs from the present study** | | | | |
| rs3817963 | 32368086 | HLA-DQA2 | 4.03E-13 | 0.925 |
| **rs9268403** | **32341472** | **HLA-DQA1** | **3.40E-07** | 1 |
| rs3763307 | 32374622 | HLA-DQA1 | 5.68E-07 | 0.222 |
| rs8180664 | 32347490 | HLA-DQA1 | 1.17E-06 | 0.223 |
| rs2076522 | 32371179 | HLA-DQA1 | 1.17E-06 | 0.223 |
| rs9268429 | 32345051 | HLA-DQA1 | 1.28E-06 | 0.907 |
| rs2076520 | 32371268 | HLA-DQB1 | 5.58E-06 | 0.889 |
| rs9268833 | 32428061 | HLA-DQA1 | 6.67E-06 | 0.72 |
|  |  |  |  |  |
| **SNPs from the previous study (1)** | | | | |
| rs2516049 | 32570399 | HLA-DQA1 | 3.48E-13 | 0.533 |
| rs477515 | 32569690 | HLA-DQB1 | 6.03E-11 | 0.533 |
| rs7754768 | 32420178 | HLA-DRB5 | 1.95E-07 | 0.285 |
| rs7192 | 32411645 | HLA-DRB5 | 2.47E-07 | 0.275 |
| rs7194 | 32412479 | HLA-DRB5 | 2.47E-07 | 0.275 |
| rs2213586 | 32413093 | HLA-DRB5 | 2.47E-07 | 0.275 |
| rs2213585 | 32413149 | HLA-DRB5 | 2.47E-07 | 0.275 |
| rs2227139 | 32413458 | HLA-DRB5 | 2.47E-07 | 0.275 |
| rs9268832 | 32427788 | HLA-DRB5 | 4.10E-07 | 0.28 |
| rs7195 | 32412538 | HLA-DRB5 | 4.61E-07 | 0.275 |
| rs2239803 | 32411832 | HLA-DQB1 | 1.18E-06 | 0.501 |
|  |  |  |  |  |
| **SNPs associated with HL** | | | | |
| rs2395185 | 32541145 | HLA-DQA2 | 1.11E-38 | 0.522 |
| rs9268542 | 32492699 | HLA-DQA2 | 2.16E-16 | 0.292 |
| rs6903608 | 32536263 | HLA-DQA1 | 1.00E-09 | 0.154 |
| rs2858870 | 32680229 | HLA-DQB1 | 3.47E-05 | 0.046 |
| rs204999 | 32217957 | HLA-DQA1 | 5.16E-05 | 0.002 |
